# Methylome-wide change associated with response to electroconvulsive therapy in depressed patients

**DOI:** 10.1038/s41398-021-01474-9

**Published:** 2021-06-05

**Authors:** Lea Sirignano, Josef Frank, Laura Kranaster, Stephanie H. Witt, Fabian Streit, Lea Zillich, Alexander Sartorius, Marcella Rietschel, Jerome C. Foo

**Affiliations:** 1 Department of Genetic Epidemiology in Psychiatry, Central Institute of Mental Health, Medical Faculty Mannheim, University of Heidelberg, Mannheim, Germany; 2 Department of Psychiatry and Psychotherapy, Central Institute of Mental Health, Medical Faculty Mannheim, University of Heidelberg, Mannheim, Germany

**Keywords:** Clinical genetics, Depression, Clinical genetics

## Abstract

Electroconvulsive therapy (ECT) is a quick-acting and powerful antidepressant treatment considered to be effective in treating severe and pharmacotherapy-resistant forms of depression. Recent studies have suggested that epigenetic mechanisms can mediate treatment response and investigations about the relationship between the effects of ECT and DNA methylation have so far largely taken candidate approaches. In the present study, we examined the effects of ECT on the methylome associated with response in depressed patients (*n* = 34), testing for differentially methylated CpG sites before the first and after the last ECT treatment. We identified one differentially methylated CpG site associated with the effect of ECT response (defined as >50% decrease in Hamilton Depression Rating Scale score, HDRS), *TNKS* (*q* < 0.05; *p* = 7.15 × 10^−8^). When defining response continuously (ΔHDRS), the top suggestive differentially methylated CpG site was in *FKBP5* (*p* = 3.94 × 10^−7^). Regional analyses identified two differentially methylated regions on chromosomes 8 (Šídák’s *p* = 0.0031) and 20 (Šídák’s *p* = 4.2 × 10^−5^) associated with ΔHDRS. Functional pathway analysis did not identify any significant pathways. A confirmatory look at candidates previously proposed to be involved in ECT mechanisms found CpG sites associated with response only at the nominally significant level (*p* < 0.05). Despite the limited sample size, the present study was able to identify epigenetic change associated with ECT response suggesting that this approach, especially when involving larger samples, has the potential to inform the study of mechanisms involved in ECT and severe and treatment-resistant depression.

## Introduction

Depression is a major contributor to global burden of disease and despite worldwide research efforts, the heterogeneous nature of the disorder makes it difficult to definitively unravel its underlying etiology and the factors influencing treatment response^1,2^. Electroconvulsive therapy (ECT) is an intervention with rapid and striking antidepressant effects and is the treatment of choice for patients with severe and treatment-resistant depression^3,4^. Studying biological changes associated with response to ECT in such a subgroup of severely depressed patients is a promising approach to gain insights into the underlying mechanisms of depression and treatment response.

DNA methylation is thought to be involved in disease pathology through its influence on gene expression and cellular function^5,6^. There is evidence that pre-treatment methylation profiles may predict likelihood of achieving remission^7^ and the literature on DNA methylation in depression has pointed to several possible genes of interest (e.g., *BDNF*, *SLC6A4*, *NR3C1*, *FKBP5*, etc. for reviews, see^7–10^) as being related to antidepressant response.

Investigating change of methylation levels during treatment may inform the biological processes underlying both depression and antidepressant response. Examining these changes in ECT patients offers an optimal research setting as: (1) treatment effects are substantial and occur soon after the intervention, and (2) ECT patients represent a subgroup of patients with the most severe form of depression. It is likely that this subgroup is not only clinically but also genetically more homogenous, especially as these patients tend to show a higher genetic burden for major depression than those with less severe forms^11^.

The few studies exploring differences in methylation related to ECT are only beginning to give insight into the factors involved. A translational study observed that the response to electroconvulsive stimulation (ECS) in rats was predicted by higher p11 promoter methylation and found this also to be the case in two human samples (*n* = 11; *n* = 65) in response to ECT^12^. Another candidate gene is brain-derived neurotrophic factor (*BDNF*) which is involved in neuroplastic changes associated with stress and depression^13,14^. Methylation differences of the promoter region of *BDNF* have been observed in many psychiatric disorders^15^, and also after use of antidepressant medication^16^. Studies in animals and humans also propose an involvement of *BDNF* in response to ECT: after ECS/ECT, alterations of *BDNF* levels have been found in rats^17^, and in humans, differences in expression of *BDNF* have been described^18,19^. Furthermore, associations between pre-treatment *BDNF* levels and ECT outcome have been described, but whether *BDNF* levels are indicative of response remains to be determined^20,21^.

Research on ECT-related changes in methylation levels has largely focused on candidate genes. However, as ECT is a non-specific treatment with widespread, yet uncertain effects on biological processes, the selection of predefined candidates, relying on prior knowledge may not give the full picture. As such, an approach investigating the whole methylome is expected to yield new and relevant insights. Few studies have made use of these technologies in ECT samples to date. Moschny et al.^22^ examined longitudinal DNA methylation before and after ECT in a small group of patients (8 responders and 4 non-responders). They did not find any significant differences in global DNA methylation, but identified eight genes potentially implicated in either ECT response or its mechanism through analysis of single probe variance, and two genes whose methylation changed during treatment course.

In the present study, we aimed to identify changes in methylation levels associated with the effects of ECT and to find potential biomarkers for antidepressant response. We obtained and compared epigenome-wide DNA methylation levels of ECT patients (*n* = 34) before and after ECT. Differentially methylated CpG sites and regions associated with response were examined. Pathway analyses were employed to search for functional pathways affected by ECT. Finally, we took a targeted look at methylation in genes which have been previously implicated in ECT response and depression-related studies.

## Materials and methods

This study was approved by the Ethics Committee (II), Medical Faculty Mannheim, University of Heidelberg. All patients provided written consent after a detailed explanation of the content of the study. All experimental procedures were performed in accordance with the Declaration of Helsinki.

### Participants

Patients (*n* = 34) diagnosed with a major unipolar depressive episode (based on International Classification of Diseases version 10, ICD-10), older than 18 years, and assigned to ECT treatment were recruited at the Department of Psychiatry and Psychotherapy at the Central Institute of Mental Health (CIMH) in Mannheim, Germany, between 2014 and 2016. Criteria for assignment to ECT were treatment-resistant depression (i.e., failure of two adequate dose-duration antidepressants from different classes, or psychotherapy in the current episode), positive experience of ECT from a previous episode, or severe depression with (a) psychotic symptoms, (b) severe suicidality, or (c) the refusal of food or fluid intake. Patients were excluded if they had any substance-use-related disorders (other than tobacco and alcohol use disorders) or a lifetime diagnosis of schizophrenia. All participants were of Caucasian descent. The patients kept the same medication regimens throughout ECT treatment. This sample is a subset of a sample reported in a previous genetic study in ECT patients^11^. Descriptive and clinical characteristics of the participants are described in Table 1.

**Table 1:** Patient demographics.

	Responders (*n* = 25)	Non-responders (*n* *=* 9)	*p-*Value (group comparison)
Sex (M/F)	16/9	2/7	0.05^a^
Smoking (NS/S)	22/3	4/5	0.02^a^
	Mean (SD)	Mean (SD)	
Age	65.8 (18.7)	55.8 (15.4)	0.16^b^
BMI	24.8 (3.9)	26.6 (5.3)	0.30^b^
Baseline HDRS	28.7 (5.4)	27.0 (6.0)	0.43^b^
Baseline HDRS (min - max)	19 - 38	21 - 41	
∆HDRS	21.1 (6.2)	6.1 (6.7)	8.21 × 10^−7b^
Number of ECT sessions	10.2 (4.7)	12.4 (6.4)	0.28^b^
Number of weeks (T1 - T2)	4.6 (1.9)	5.5 (2.6)	0.28^b^
ECT sessions per week	2.2 (0.1)	2.2 (0.1)	0.45^b^

*M* male, *F* female, *NS* non-smoker, *S* smoker, *BMI* body mass index, *HDRS* Hamilton Depression Rating Scale, *ECT* electroconvulsive therapy, *SD* standard deviation.

^a^Fisher’s exact test.

^b^*t*-test.

### Depression and DNA methylation assessment

The 21-item version of the Hamilton Depression Rating Scale (HDRS) was administered and blood was collected from patients prior to the first (T1) and after the last (T2) session of ECT (average sessions: 10.82, range: 5–25 sessions). T2 was between 1 and 7 days after the last ECT session. Time of collection was kept constant between 8:30 and 9:30 a.m. at both timepoints to keep it close to the clinical interview conducted around the same time, as well as preclude any potential confounding effects arising from ward routines and circadian fluctuations^23,24^. Standard procedures for extraction and processing were followed. DNA extraction was performed using the Chemagic Magnetic Separation Module 1 (Chemagen Biopolymer-Technologie AG; Baesweiler, Germany). All genomic DNA samples were stored at −20 °C prior to analysis. Epigenome-wide DNA methylation was measured using the Illumina Infinium Methylation EPIC array (>850,000 CpG sites). The arrays were processed at the Genome Analysis Center at the HelmholtzZentrum München and Max-Planck-Institute for Psychiatry in Munich, Germany.

### ECT treatment

ECT was conducted with a Thymatron IV device (Somatics, LLC. Lake Bluff, IL, USA). Anesthetic drugs used were: s-ketamine (~1.0 mg/kg)^25,26^ and succinylcholine for muscle relaxation (~1.0 mg/kg). Seizure thresholds were titrated at the initial session; dosing in subsequent sessions was given at >2.5 above this threshold. If patients did not show a clinical improvement or seizures were insufficient, energy used was increased. Patients received 2–3 ECT sessions per week. All patients started with unilateral stimulation with the possibility to change to bilateral stimulation at the discretion of the ECT supervisor.

### Statistical analysis

Data processing, quality control (QC) and other statistical analyses were performed using R (versions 3.4.4 and 3.6.3) analysis software (https://cran.r-project.org/).

#### Data preprocessing, QC, and filtering

Methylation values were extracted using an updated version of the pipeline indicated in^27^, adapted in-house for use with the Illumina Infinium EPIC array. Illumina background correction was applied to all intensity values. A detection *p*-value threshold of *p* < 10^−16^ was used and intensity values with detection *p* ≥ 10^−16^ were designated as missing data. The proportion of missing data points was determined, allowing the calculation of sample and CpG site-specific call rates. Samples with insufficient DNA quality as denoted by a call rate of <95% were excluded. Intensity values were quantile normalized for each of the six probe types present on the array separately. Intensity values were converted to methylation beta values according to the manufacturer’s recommendation. White blood cell fractions were estimated according to^28^. Five of the six resulting estimates were subsequently included as covariates in downstream analyses to control for influences of cell type distribution on DNA methylation. The estimate for granulocytes showed the highest variance inflation factor (VIF) and was omitted to avoid collinearity issues. CpG sites were filtered by removing cross-hybridizing probes, probes with high missing rate (>0.02), and probes linked to X- and Y-chromosomes. Correction for batch effects and other technical parameters was done by performing a principal component analysis on internal control probe intensity values and including the first 10 extracted principal components (PCs) as covariates in the downstream analyses. Prior to analysis, all methylation beta values were logit transformed (base 2) to M-values, which were used in downstream analysis as recommended in^29^.

#### Differentially methylated single CpG sites

Association testing of methylation M-values for each CpG site was done using a mixed linear model approach as implemented in the Limma R package. Participant ID was used as a blocking factor, and estimated cell fractions, 10 control probe PCs, age, sex, and smoking status were included as additional covariates to adjust for confounding factors. It was observed that sex was highly correlated with the 5th control probe PC, thus this PC was not included in the statistical models to avoid collinearity issues.

The main effects of interest specified included: (1) response (responders vs. non-responders), (2) timepoint (change after intervention), and (3) the interaction between timepoint and response (the difference in change between response groups). Models were calculated with response specified both as a binary (>50% decrease in HDRS score) and continuous variable (i.e., change in HDRS score, ΔHDRS).

In addition, we examined the relationship between baseline (T1) methylation and response (both binary and continuous) in additional models.

Significance was defined as false discovery rate (FDR) *q* < 0.05. Results at a suggestive threshold of *p* < 10^−5^ were also reported.

#### Differentially methylated regions

Differentially methylated region (DMR) analysis was performed on the results of the above analyses using the comb-p package^30^. Comb-p parameters were specified as: seed *p*-value = 0.001 and a maximum distance between probes of 500 base pairs. These parameters follow those used in previous studies in the field^31,32^ and results from simulation experiments^33^.

#### Pathway analysis

A Gene Ontology (GO) enrichment analysis was performed on the results of the different models using the missMethyl (v1.12.0) R package. We examined CpG sites at the suggestive threshold of 1 × 10^−5^.

#### Targeted examination of methylation change in candidate CpG sites

Change in methylation of candidate genes from the literature was examined in an exploratory search. First, we selected: (1) candidates implicated in reviews of DNA methylation and antidepressant medication, i.e., *BDNF*, *MAOA*, *SLC6A2*, *SLC6A4*, *HTR1A*, *HTR1B*, *IL6*, *IL11*^7^; *SLC6A4*, *NR3C1*, *FKBP5*, and *OXTR*^8^; and (2) candidates specific to ECT, i.e., *S100A10* (p11)^12^, *RNF175*, *RNF213*, *TBC1D14*, *TMC5*, *WSCD1*, *AC018685.2*, *AC098617.1*, *CLCN3P1*, *AQP10*, and *TRERF1*^22^.

Autosomal CpG sites which were annotated to these candidate genes in the UCSC Genome Browser NCBI curated RefSeq (retrieved: August 10, 2018) were extracted from the results of the single site analyses above (for each variable of interest in both binary and continuous models). Furthermore, to examine their predictive value in our sample, association between baseline methylation and response was also examined. A secondary FDR correction was applied to the list of all candidate CpG sites to control for false positives.

## Results

Descriptive statistics of the sample are shown in Table 1. In the sample analyzed, using binary criteria, (defined as decrease of HDRS score of more than 50%) 25 were responders and 9 were non-responders to ECT. Levene’s test found no significant differences between group variances. Briefly, as also reported for the total sample in^11^, binary response to ECT was positively correlated with sex (being male) while continuous response (ΔHDRS score) was also associated with male sex and positively correlated with increased age.

### Single CpG site analysis

#### Binary response

In the binary response model, one significantly differentially methylated site (*q* < 0.05), cg10005358, mapped to *TNKS*, was observed as an effect of response. Eight sites reached a suggestive threshold of *p* < 1 × 10^−5^ (see Table 2). No significantly differentially methylated CpG sites were observed for the effect of timepoint (at *p* < 1 × 10^−5^; 22 CpG sites), or interaction effect (at *p* < 1 × 10^−5^; 12 CpG sites). Several CpG sites annotated to the same gene appeared among the top hits of these effects of interest (see Tables 2 and S1.1–S1.3, e.g., *TNKS*, *PCM1*, *RAPGEF2*, *RAB21*; all suggestive at *p* < 1 × 10^−5^).

**Table 2:** Top 10 differentially methylated CpG sites associated with binary response and ΔHDRS.

CpG	CHR	Base pair position	*p-*Value	FDR	Annotated genes
Binary response					
cg10005358	8	9505300	7.2 × 10^−8^	0.0498	*TNKS*
cg22813821	12	72148853	2.2 × 10^−6^	0.5940	*RAB21*
cg11062168	15	35262789	2.6 × 10^−6^	0.5940	*AQR*
cg12305855	4	160216262	5.1 × 10^−6^	0.6072	*RAPGEF2*
cg19869734	2	107154571	9.8 × 10^−6^	0.6072	
cg08133350	19	50321326	9.9 × 10^−6^	0.6072	*MED25*
cg23870282	3	72897792	1.0 × 10^−5^	0.6072	*SHQ1*
cg00101693	10	70715578	1.0 × 10^−5^	0.6072	*DDX21*
cg23367665	1	231414306	1.1 × 10^−5^	0.6072	
cg00511318	17	56406260	1.1 × 10^−5^	0.6072	*TSPOAP1;TSPOAP1-AS1*
ΔHDRS					
cg01294490	6	35656906	4.5 × 10^−7^	0.3106	*FKBP5*
cg10515948	2	242674491	1.3 × 10^−6^	0.4558	*D2HGDH*
cg02936535	17	7514491	3.8 × 10^−6^	0.7870	*FXR2*
cg11385008	2	11621166	6.1 × 10^−6^	0.7870	
cg16377817	5	170845627	8.2 × 10^−6^	0.7870	*FGF18*
cg06668695	15	80213874	8.6 × 10^−6^	0.7870	*ST20-MTHFS;ST20;ST20-AS1*
cg08790000	11	67255752	9.0 × 10^−6^	0.7870	*AIP*
cg16306546	21	44183372	1.0 × 10^−5^	0.7870	*PDE9A*
cg19307750	1	241372556	1.1 × 10^−5^	0.7870	*RGS7*
cg03611990	6	96980568	1.2 × 10^−5^	0.7870	*UFL1*

*CpG* cytosine-phosphate-guanine, *CHR* chromosome, *FDR* false discovery rate.

#### Continuous response (ΔHDRS)

In the continuous response model, no effects yielded significantly differentially methylated CpG sites at *q* < 0.05. At a suggestive threshold of *p* < 1 × 10^−5^, 7, 9, and 5 differentially methylated CpG sites were observed for the effects of ΔHDRS, timepoint, and ΔHDRS × timepoint, respectively (see Table 2 and Tables S2.1–S2.3). *FKBP5* (CHR 6: cg01294490) was the top hit for both effect of ΔHDRS (*p* = 4.46 × 10^−7^) and effect of interaction of ΔHDRS × timepoint (*p* < 3.94 × 10^−7^). *FXR2* (CHR 17: cg02936535) was also observed among the top hits for all effects of interest (ΔHDRS *p* = 3.79 × 10^−6^; timepoint *p* = 6.78 × 10^−6^; ΔHDRS × timepoint *p* = 7.38 × 10^−6^).

#### Baseline methylation and response

Methylation at baseline was not significantly associated with either binary or continuous response. At a suggestive threshold of *p* < 1 × 10^−5^ baseline methylation was associated with binary response at 9 CpG sites and with ΔHDRS at 6 CpG sites (see Tables S3.1 and S3.2).

### Differentially methylated region analysis

**Fig. 1:**
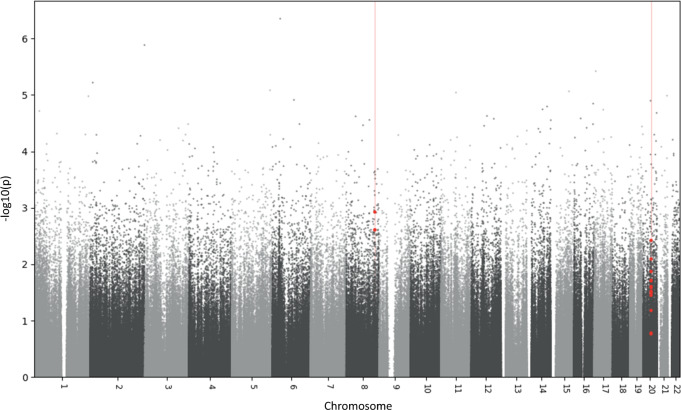
Manhattan plot of differentially methylated regions for ΔHDRS. Significantly differentially methylated regions associated with the effect of ΔHDRS are indicated in red (chromosomes 8 and 20).

Two DMRs were identified as associated with effect of ΔHDRS in the continuous response model. One significant DMR on chromosome 8 was identified (3 probes, Šídák’s corrected *p* = 0.0031) and another on chromosome 20 (13 probes, Šídák’s corrected *p* = 4.2 × 10^−5^). The DMR on chromosome 8 (CHR 8: 127568854-127569023) is located in the *LRATD2* (*FAM84B*) gene, while the chromosome 20 DMR (CHR 20: 36148620-36148861) is located in the *BLCAP* gene and in the promoter region of *NNAT* (791 base pairs upstream of the transcription start site, TSS). Two other regions on chromosomes 14 and 19 were nominally significant but did not remain significant after Šídák correction (see Table 3 and Fig. 1).

**Table 3:** Differentially methylated regions associated with ΔHDRS.

CHR	Base pair start - end	Min *p-*value	Number of probes	Šídák’s *p-*value	Annotated genes
20	36148620 - 36148861	2.4 × 10^−4^	13	4.2 × 10^−5^	*BLCAP, NNAT*
8	127568854 - 127569023	2.4 × 10^−4^	3	0.0031	*LRATD2 (FAM84B)*
19	39402922 - 39402937	3.0 × 10^−4^	3	0.4673	*CCER2*
14	91720372 - 91720373	7.9 × 10^−4^	1	1	*GPR68*

*CHR* chromosome.

Analysis of the results from timepoint and timepoint × ΔHDRS models, as well as all binary response models, did not identify significant DMRs.

### Pathway analysis

No significant pathways were observed in any of the models.

### Candidate analysis

In the binary model, at nominal significance (*p* < 0.05 uncorrected), 43, 37, and 36 CpG sites were associated with response, timepoint, and response × timepoint interaction, respectively. The top 3 candidate CpG sites associated with binary response were: *FKBP5* (cg01294490, *p* = 8.74 × 10^−5^), *BDNF-AS* (cg02386995, *p* = 1.51 × 10^−4^), and *NR3C1* (cg23273257, *p* = 0.0012), but no CpG site tested survived secondary FDR correction for multiple testing.

In the continuous response model, 41, 43, and 42 CpG sites were nominally associated with ΔHDRS, timepoint and ΔHDRS × timepoint interaction, respectively. The top 3 candidate CpG sites associated with continuous response were, *FKBP5* (cg01294490, *p* = 4.46 × 10^−7^), *BDNF* (cg15710245, *p* = 0.0033), and *BDNF-AS* (cg09878183, *p* = 0.0034). The site annotated to *FKBP5* survived the secondary correction for multiple testing (*q* = 0.0004).

## Discussion

The present study examined treatment-associated changes of DNA methylation levels in 34 patients in an epigenome-wide manner. By investigating the relationship between response status and change in methylation levels, this study identified several potential CpG sites involved in ECT response and outlines potential differences between response groups.

The top CpG site associated with binary response is located in *TNKS*, which is a protein-coding gene associated with blood pressure, alcohol consumption, implicated in cancer pathology, and involved in various processes such as the Wnt signaling pathway, telomere length, and vesicle trafficking^34^. Telomere length, a marker associated with aging is also known to be associated with psychiatric disorders^35^ including major depressive disorder^36^, as well as depressive symptoms^37^. In several genome-wide association studies (GWASs) of depression-related traits, *TNKS* was found to be associated with (*p* = 7.68 × 10^−10^)^38^, bipolar disorder (*p* = 3 × 10^−6^)^39^, and positive affect (*p* = 0.0003)^40^.

Among the top 5 CpG sites suggestively associated with binary response, genes associated with processes such as cell adhesion, cell growth, apoptosis in malignant tumors, protein metabolism (*RAB21*)^41^, and signaling in glucose metabolism (*AQR*)^42^ were found. *RAB21* was previously reported to be associated with remission (*p* = 0.0103) in a GWAS of selective serotonin reuptake inhibitors in MDD patients^43^ and *AQR* as related to neuroticism (*p* = 9.58 × 10^−8^) and worry (*p* = 2.06 × 10^−6^)^44^, a well-known symptom in depression. *RAPGEF2*, a protein-coding gene suggested to be involved in signal transmission, in BDNF receptor pathway signaling^45^, in schizophrenia^46^, and is found to be a target for regulated miRNAs in MDD^47^. *PCM1*, located in a chromosomal region on 8p, which has been implicated in various neuropsychiatric disorders including schizophrenia and depression^48^, is a protein-coding gene critical for cell division, and is involved in the proliferation and neurogenesis of neuroprecursors^49^.

The top site in the continuous response analysis was located in *FKBP5*, a gene which is known to be an important endogenous regulator of the stress hormone system possibly linked to stress-related psychiatric disorders such as depression^50^. *FKBP5* demethylation resulting from childhood trauma has been linked to long-term stress hormone system deregulation and effects on immune function and brain areas associated with stress regulation^51^. Depressive phenotypes are shown to be associated with the age-related decrease in *FKBP5* methylation^52^. Altered epigenetic and genetic *FKBP5* regulation may contribute to stress-related disease risk. Findings related to *FKBP5* have pointed to it as important in the interaction with environment in stress-related disorders such as major depression^53^. The present study found an association between methylation in a CpG site in *FKBP5* and the reduction in patients’ HDRS scores. Although the CpG site identified here has not been associated with antidepressant response in previous studies, the findings support *FKBP5* as an important gene requiring further investigation in the present context.

Among the top 5 CpG sites suggestively associated with continuous response were CpG sites annotated to *D2HGDH* and *FXR2*. *D2HGDH* encodes for the enzyme D-2-hydroxyglutarate dehydrogenase, and is suggested to be downregulated in depressed patients during remission^54^. Proteins of the FXR family have commonly been reported in autism spectrum disorders, and evidence from GWASs in mood disorders and schizophrenia suggests that Fragile X mental retardation syndrome-related proteins are involved in the development of mental disorders^55^.

Two DMRs associated with continuous response were identified. The DMR on chromosome 8 lies in *LRATD2 (FAM84B)*, which is known to be involved in gastric and prostate cancer^56,57^. In a recent large genome-wide gene-environment analysis, its paralog, *LRATD1*, was observed to be associated with unipolar depression and response to trauma exposure^58^. The DMR on chromosome 20 is located in *BLCAP* and in the promoter region of *NNAT*. *BLCAP* encodes a protein that regulates cell proliferation and reduces cell growth by stimulating apoptosis^59^, and *NNAT* is involved in brain development and neuronal differentiation^60^. Together with the single site results, these findings are in line with previous works in the field of ECT; alterations in mechanisms such as neurogenesis and neuroinflammatory immune response are proposed to be among the mechanisms of ECT action^61–63^.

Several candidate genes proposed in the literature were found harboring CpG sites with nominally significant changes between T1 and T2, and T1 methylation values for a number of them were also nominally associated with response (both binary and continuous) (see Tables S4.1–S6.2); the roles they play remain unclear. The present results appear to lend support to previous research which has suggested the importance of these candidates but these results are preliminary and further investigation is warranted. Also, the identification of *FKBP5* in the present study suggests that future research should assess and control for factors such as childhood trauma and stress^51,64^.

This study had several limitations. Although the largest study to date, the present sample size was limited, and it is expected that future studies using a similar approach in larger samples will be able to further clarify our results. Sample size notwithstanding, we identified a single significantly differentially methylated CpG site, as well as some suggestive ones which need further investigation. While we assessed methylation levels in whole blood, ECT is applied to the brain; both central and peripheral mechanisms may be affected by the global nature of the treatment and care should be taken with the interpretation of these findings^65^. The possible effect of anesthesia and pharmacotherapy is a potential confounding factor in methylation studies. However, medication in each patient was kept constant during the ECT course, and there were no differences between patients regarding anesthesia administration or treatment dosage. Therefore, the observed changes are unlikely to have resulted from these medications.

The genes implicated in our findings have been previously involved in the etiology of depression and treatment response, but confirmation in larger samples is needed. Multi-center approaches and collaborative efforts could help in obtaining the sample sizes required to allow a more robust characterization of ECT response and give insights into the biological processes underlying the striking antidepressant effects of ECT.

## Supplementary information

Supplementary tables.
